# Up-regulation of circ_LARP4 suppresses cell proliferation and migration in ovarian cancer by regulating miR-513b-5p/LARP4 axis

**DOI:** 10.1186/s12935-019-1071-z

**Published:** 2020-01-06

**Authors:** Wumei Lin, Haiyan Ye, Keli You, Le Chen

**Affiliations:** Department of Gynecology, Guangdong Provincial People’s Hospital, Guangdong Academy of Medical Sciences, No.106 Zhongshan 2 Road, Guangzhou, 510080 Guangdong China

**Keywords:** OC, circ_LARP4, miR-513b-5p, ceRNA, LARP4

## Abstract

**Background:**

Ovarian cancer (OC) is a common fatal malignant tumor of female reproductive system worldwide. Growing studies have proofed that circular RNAs (circRNAs) engage in the regulation of various types of cancers. However, the underlying biological functions and effect mechanism of circular RNA_LARP4 (circ_LARP4) in OC have not been explored.

**Methods:**

Quantitative real-time polymerase chain reaction (qRT-PCR) analysis was used to detect the expression of circ_LARP4 in OC cells. The function of circ_LARP4 was measured by cell counting kit-8 (CCK-8), colony formation assay and transwell assay. RNA immunoprecipitation (RIP) assay and luciferase reporter assays assessed the binding correlation between miR-513b-5p and circ_LARP4 (or LARP4).

**Results:**

The expression of circ_LARP4 in OC cells was much lower than that in human normal ovarian epithelial cells. Overexpressing circ_LARP4 impaired cell proliferation, invasion and migration abilities. Circ_LARP4 worked as a competing endogenous RNA (ceRNA) to sponge miR-513b-5p. Furthermore, LARP4 was indirectly modulated by circ_LARP4 as the downstream target of miR-513b-5p, as well as the host gene of circ_LARP4.

**Conclusion:**

Circ_LARP4 could hamper cell proliferation and migration by sponging miR-513b-5p to regulate the expression of LARP4. This research may provide some referential value to OC treatment.
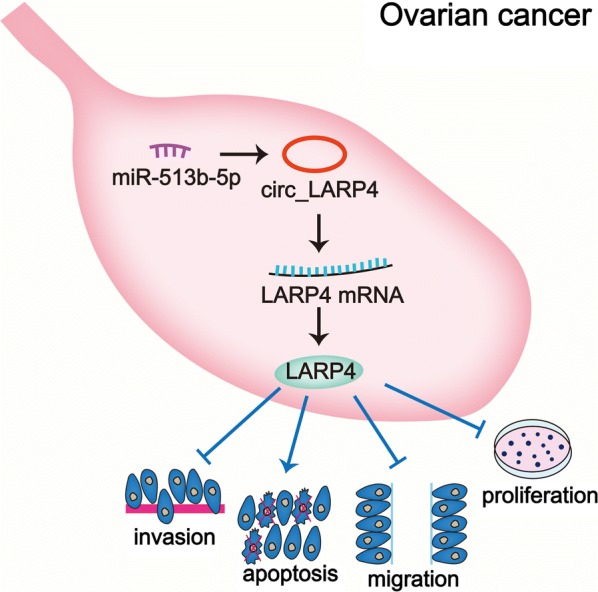

## Background

Ovarian cancer (OC) is a highly fatal gynecologic malignant tumor of female reproductive system worldwide [1, 2]. This disease has no symptom usually and is hardly to verify in the early stage [3]. More than 70% of cases are diagnosed in the advanced stage. The 5-year survival rate of disease is around one-fifth to one-third [4, 5]. Consequently, the key molecule of forecasting and diagnosing OC is necessary to be determined. Nowadays, the main treatments for OC are surgery and chemotherapy. However, the recurrence rate is high, drug resistance is strong and the prognosis is unfavorable. Especially for patients with late, the 5-year survival rate of ovary is less than 40 percent [6]. Improving the current situation needs susceptive early detection index and new therapeutic targets [3, 7].

Recently, circular RNAs (circRNAs) were estimated as biological marker of certain types of tumors [8]. Highly expressed circRNA UBAP2 was dramatically related to the progression and prognosis of human osteosarcoma (HuOs) [9]. CircRNA-MYLK significantly promoted the process of bladder cancer [10]. Growing Evidence has shown that some circular RNAs act as a sponge for microRNAs (miRNAs) modulating gene transcription. Besides, circRNAs are able to engage in the RNA binding protein (RBP) interactions of tumorigenesis [11, 12]. For instance, ciRS-7 being the sponge for miR-7 regulated the expression of multiple oncogenes [13]. CircHIPK3 inhibited cell proliferation abilities of many cancer genes as the sponge for miR-124 [14]. Meanwhile, circular RNA_LARP4 (circ_LARP4) was assessed correlated to pathological staging and unfavorable prognosis of gastric cancer [15]. In addition, circ_LARP4 was attested lowly expressed in OC [16]. However, the biological functions and effect mechanism of circ_LARP4 in OC have not been studied further.

Hence, we investigated the role of circ_LARP4 in OC and its function of modulating miRNA/messenger RNA (mRNA) axis to regulate the biological behavior of OC cells.

## Materials and methods

### Cell culture

Human ovarian carcinoma cells (SKOV3, A2780, SW626, OVCAR3 and OVCAR4) and human ovarian surface epithelial cell (HOSEpiC) were purchased from American Type Culture Collection (ATCC; Manassas, VA, USA). At 37 °C, cells were propagated with RPMI 1640 medium (Gibco, Rockville, MD, USA) supplementing 10% FBS (Gibco), 100 U/mL penicillin and 100 mg/mL streptomycin (Gibco) in a humidified atmosphere with 5% CO_2_.

### Cell transfection

The pcDNA3.1(+) CircRNA Mini Vector targeting circ_LARP4 (OE-circ_LARP4) and the empty vector, the pcDNA3.1 vector targeting LARP4 (OE-LARP4) and the empty vector, as well as specific shRNA against LARP4 (sh-LARP4) and its corresponding NC (sh-NC), were attained from Genechem (Shanghai, China). Moreover, miR-513b-5p mimics and NC mimics were attained from GenePharma (Shanghai, China). These mentioned plasmids were separately transfected into SKOV3 or A2780 cells by use of Lipofectamine 3000 (Invitrogen, Carlsbad, CA, USA).

### qRT-PCR

TRIzol reagent (Invitrogen) was applied for isolation of total RNA. Reverse transcription reactions were undertaken with the 4× Reverse Transcription Master Mix kit (EZBioscience, Shanghai, China). qRT-PCR were carried out utilizing FastStart Universal SYBR Green Master (Roche, Mannheim, Germany). U6 or GAPDH was applied to normalize results. Gene expression levels were quantified using 2^−∆∆CT^ method.

### Actinomycin D (ActD) and RNase R treatment

For ActD treatment, through addition of 2 mg/ml Actinomycin D (Sigma-Aldrich, St. Louis, MO, USA), transcription was prevented. For RNase R treatment, total RNA was incubated with 3 U/μg of RNase R (Epicentre Technologies, Madison, WI, USA). RNA expression levels were assayed with qRT-PCR.

### Cell viability and colony formation assays

Cell viability assay was undertaken with cell counting kit 8 (CCK8; Dojindo, Tokyo, Japan). For the purpose of assessing cell colony formation efficiency, transfected SKOV3 or A2780 cells were plated in 6-well plates. After 14 days of growth, colonies were washed thrice in PBS (Sigma-Aldrich) and dyed in crystal violet (Sigma-Aldrich) for counting.

### Western blot

Western blot was implemented on the basis of prior description [17]. Primary antibodies against Bax (ab32503), Bcl-2 (ab32124), Cleaved caspase-3 (ab2302), Total caspase-3 (ab13847), Cleaved caspase-9 (ab2324), Total caspase-9 (ab32539), MMP2 (ab37150), MMP7 (ab5706), MMP9 (ab38898), LARP4 (ab241489) and GAPDH (ab245356) applied were purchased from Abcam (Cambridge, USA).

### Migration and invasion assays

Cell invasion ability was explored with 24-well Transwell chambers (Corning Costar, Cambridge, MA, USA) containing a Matrigel-coated membrane. Transfected SKOV3 or A2780 cells were inoculated in the upper chambers. Then, medium with 10% FBS was added to the lower chambers. Upon incubation for 24 h, non-invasive cells were removed by use of a cotton swab and invasive cells were fixed by methanol (Sigma-Aldrich), followed by stained in crystal violet and dried, and finally photographed under a microscope (Olympus, Tokyo, Japan). Cell migration ability was assessed in a similarity approach without the Matrigel coating.

### Subcellular fractionation

Subcellular isolation of RNAs in SKOV3 or A2780 cells was implemented via a Cytoplasmic and Nuclear RNA Purification Kit (Norgenbiotek, Thorold, ON, Canada) as per its protocols. Next, cytoplasmic and nuclear fractions were explored by qRT-PCR.

### Fluorescence in situ hybridization (FISH)

Alexa Fluor 555-labeled circ_LARP4 probes were acquired from RiboBio (Guangzhou, China). Signals of probes were verified by the Fluorescent in Situ Hybridization Kit (RiboBio). In the end, images were captured by a fluorescence microscope (Nikon, Tokyo, Japan).

### Luciferase reporter assays

LARP4 promoter was sub-cloned into pGL3 reporter vector (Invitrogen) to form pGL3-LARP4 promoter which was co-transfected into SKOV3 or A2780 cells with OE-circ_LARP4 or the empty vector. Circ_LARP4-WT/Mut or LARP4-WT/Mut was sub-cloned into pmirGLO dual-luciferase plasmid (Promega, Madison, WI, USA) to construct pmirGLO-circ_LARP4-WT/Mut or pmirGLO-LARP4-WT/Mut which was co-transfected with miR-513b-5p mimics or NC mimics into SKOV3 or A2780 cells. Luciferase activities were examined through Dual-Luciferase Reporter Assay System (Promega).

### RNA pull-down

SKOV3 or A2780 cell lysate was incubated for 2 h with the biotinylated circ_LARP4 probe and control probe (GenePharma) adding M-280 Streptavidin magnetic beads (Invitrogen). Upon washing, the RNA complex bound to the beads was eluted, followed by extracted using a RNeasy Mini Kit (Qiagen, Hilden, Germany) and assayed by qRT-PCR.

### RNA immunoprecipitation (RIP)

The EZ-Magna RIP RNA-binding protein immunoprecipitation kit (Millipore, Bedford, MA, USA) was utilized for implementing RIP. SKOV3 or A2780 cell lysate were incubated using RIP buffer containing magnetic beads conjugated to anti-Ago2 antibody (Abcam) or anti-IgG antibody (Abcam). Upon purification of immunoprecipitated RNA, qRT-PCR was accordingly conducted.

### Statistical Analysis

Data were elicited with triplicate value and shown as mean  ±  SD. Results were assayed statistically via one-way ANOVA or student *t* test utilizing SPSS software (SPSS, Chicago, IL, USA). P < 0.05 was considered statistically significant.

## Results

### Circ_LARP4 expression is markedly down-regulated in OC cell lines

**Fig. 1:**
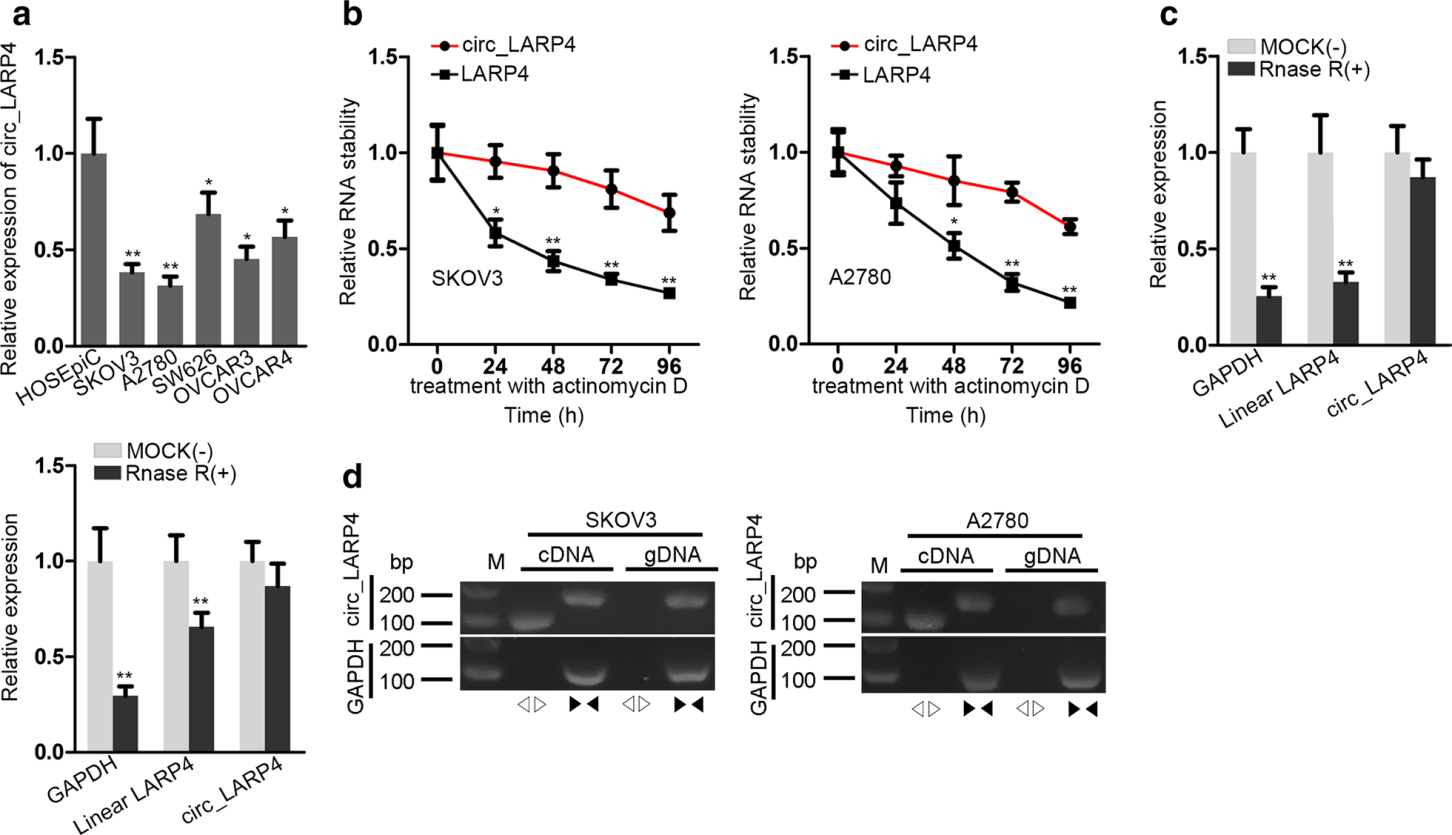
Circ_LARP4 expression is markedly down-regulated in OC cell lines. **a** Utilizing quantitative real-time polymerase chain reaction (qRT-PCR) to assess the expression of OC cell lines (SKOV3, A2780, SW626, OVCAR3, OVCAR4) and normal ovarian epithelial cells (HOSEpiC). **b** RNA expression of circ_LARP4 and LARP4 in two cells (SKOV3 and A2780) treated with actinomycin D was tested by qRT-PCR analysis. **c** The qRT-PCR analysis was used to the expression of circ_LARP4 and linear LARP4 with adding RNase R. **d** Circ_LARP4 expressions in cDNA and gDNA was measured by qRT-PCR analysis. All results were displayed as the mean ± SD. *P < 0.05, **P < 0.01

Circ_LARP4 was assessed correlated to pathological staging and unfavorable prognosis of gastric cancer [15]. In addition, low expression of circ_LARP4 was revealed in OC [16]. However, the biological functions and effect mechanism of circ_LARP4 in OC have not been studied further. Hence, at first step, we utilized qRT-PCR analysis to detect circ_LARP4 expression in OC cell lines (SKOV3, A2780, SW626, OVCAR3, OVCAR4) and human normal ovarian epithelial cells (HOSEpiC). As illustrated in Fig. 1a, circ_LARP4 expression in OC cell lines was much lower than that in normal group. For the following observation, we singled out two cell lines with lower expression of circ_LARP4, SKOV3 and A2780. Besides, by treatment with actinomycin D (a transcription suppressor), transcription half-life of circ_LARP4 was strikingly longer than that of LARP4. The result illustrated that circ_LARP4 stability was much higher than linear RNA LARP4 (Fig. 1b). Simultaneously, Fig. 1c also showed that circ_LARP4 was less susceptible to digestion caused by RNase R exonuclease by comparison with linear RNA LARP4. Figure 1b and c both proofed that circ_LARP4 had stronger stability as a circRNA. Further, circ_LARP4 was amplified in cDNA by divergent primers instead of in gDNA, which implied the loop structure of circ_LARP4 (Fig. 1d). Altogether, circ_LARP4 has relative lower expression in OC cell lines. It may be a tumor inhibitor in the process of OC cells.

### Up-regulating circ_LARP4 hampers cell proliferation and migration of OC cell lines

**Fig. 2:**
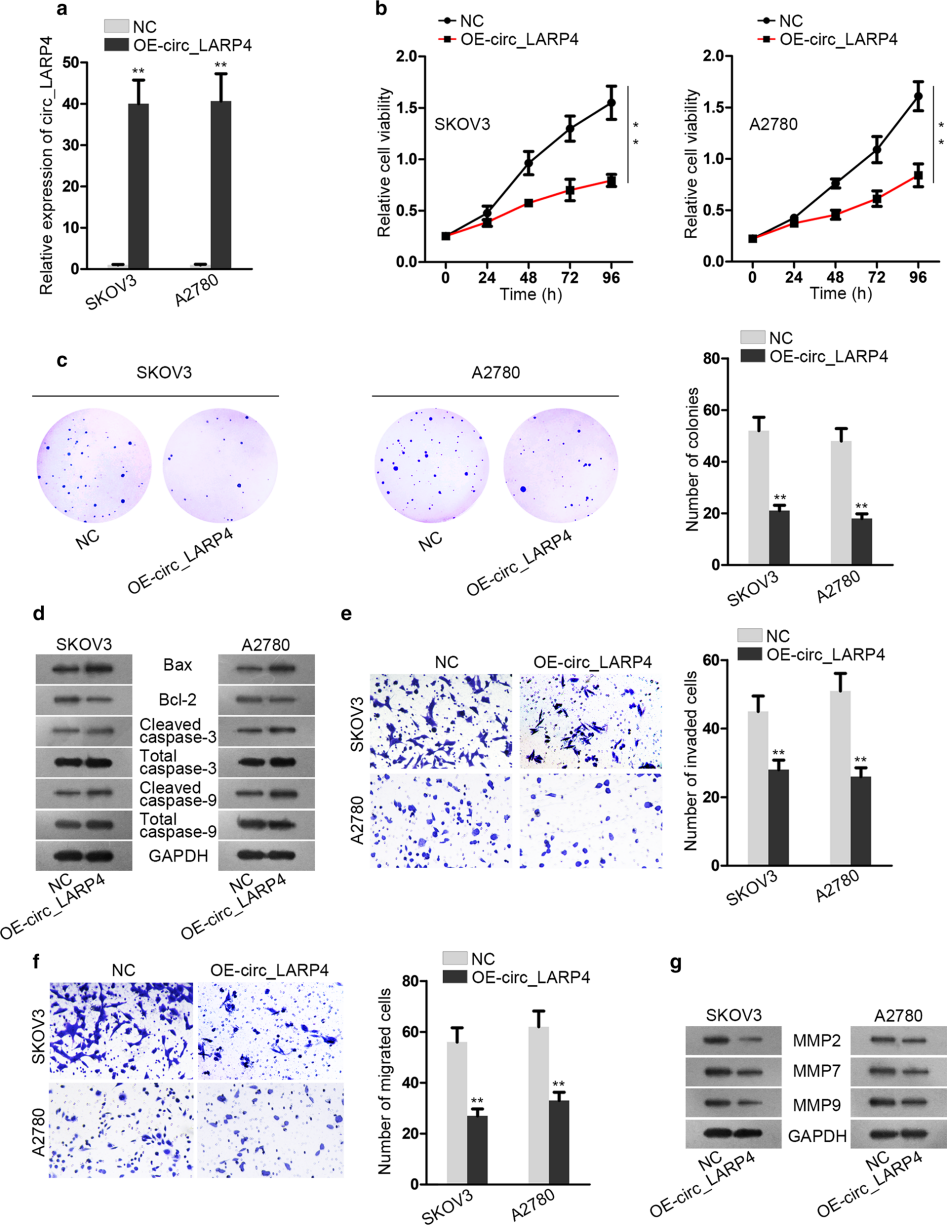
Up-regulating circ_LARP4 hampers cell proliferation and migration of OC cell lines. **a** The qRT-PCR analysis was employed to determine the expression of circ_LARP4 with increasing circ_LARP4. **b** Cell counting kit-8 (CCK-8) assay was applied to detect cell viability under circ_LARP4 overexpression (OE-circ_LARP4). **c** Colony formation assay was carried out to estimate cell proliferation ability with OE-circ_LARP4. **d** Implementing western blot assay to detect apoptosis related protein level (Bax, Bcl-2, Cleaved caspase-3, Total caspase-3, Cleaved caspase-9 and Total caspase-9) under the condition of OE-circ_LARP4. **e**, **f** Transwell assay was conducted to test cell invasion and migration capacities. **g** Invasion and migration related protein levels (MMP2, MMP7 and MMP9) were measure by Western blot assay. All data were performed as the mean ± SD. **P < 0.01

To proof the prediction about the inhibition effect of circ_LARP4 on OC progression, OE-circ_LARP4 was transfected into SKOV3 and A2780 cells. As shown in Fig. 2a, relative expression of circ_LARP4 in both cells was significantly elevated after transfection with OE-circ_LARP4. By cell counting kit-8 (CCK-8) assay, we recognized that cell viability abated under the condition of circ_LARP4 overexpression (Fig. 2b). Besides, cell formation efficiency was also decreased by increasing circ_LARP4 expression (Fig. 2c). Figure 2d and Additional file 2: Figure S2A displayed the alteration of apoptosis-related proteins expression. After circ_LARP4 expression was upregulated in SKOV3 and A2780 cells, the protein expression of Bax, Cleaved caspase-3 and Cleaved caspase-9 was increased apparently, whereas that of Bcl-2 was decreased inversely. The result manifested that overexpressing circ_LARP4 enhanced cell apoptosis ability. Furthermore, transwell assay attested that cell invasion and migration capabilities were impaired by adding circ_LARP4 to SKOV3 and A2780 cells (Fig. 2e, f). Additionally, the protein levels of migration and invasion correlated proteins (MMP2, MMP7 and MMP9) were cut down significantly which could be inferred from Fig. 2g and Additional file 2: Figure S2B. To sum up, up-regulating circ_LARP4 expression inhibits cell proliferation, migration and invasion whereas induces cell apoptosis.

### Circ_LARP4 up-regulates its host gene LARP4 after transcription

**Fig. 3:**
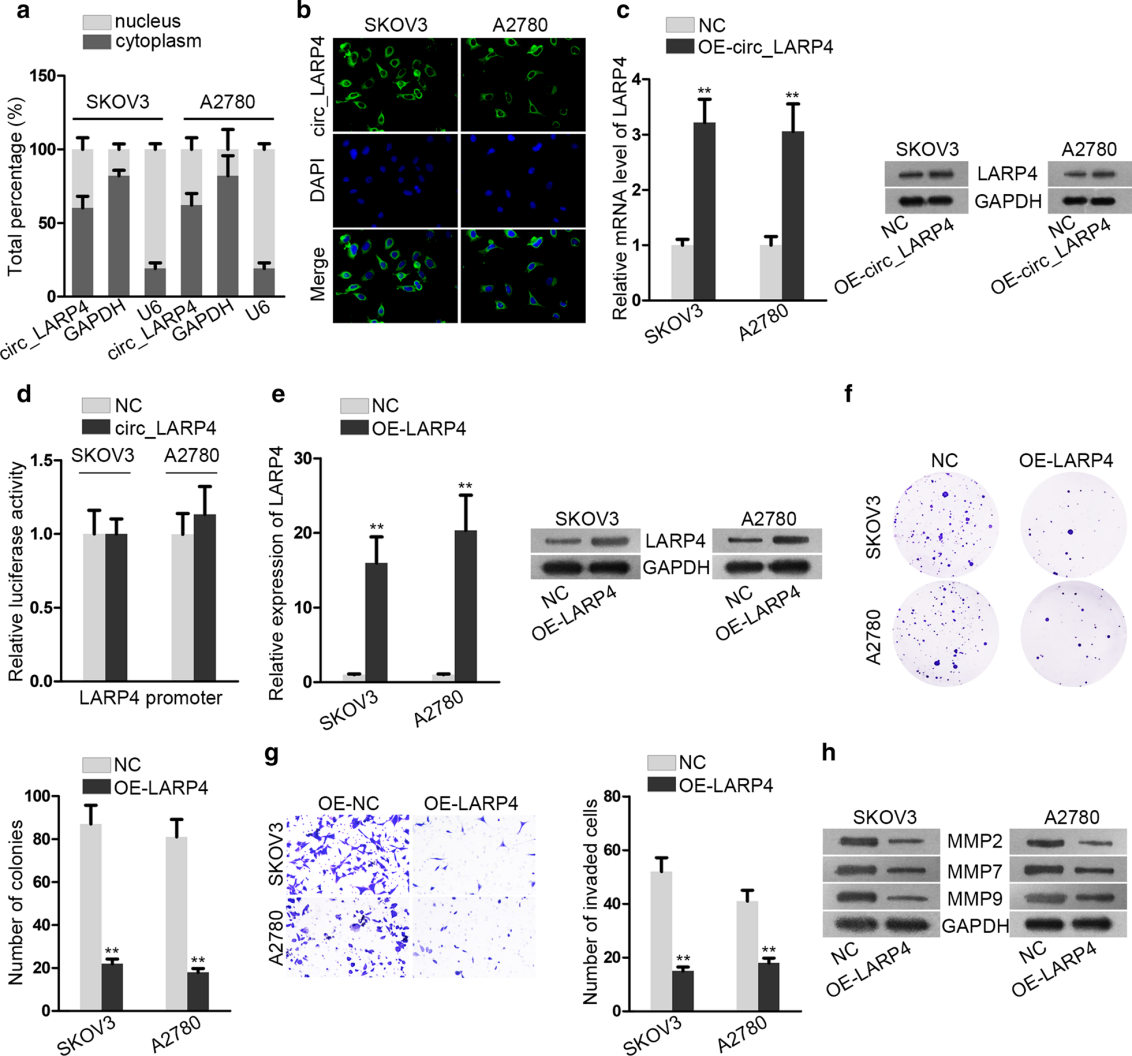
Circ_LARP4 up-regulates its host gene LARP4 after transcription. **a**, **b** Subcellular fractionation assay and fluorescence in situ hybridization (FISH) were performed to determine that circ_LARP4 was located in cytoplasm. **c** The expression and protein level of LARP4 after increasing circ_LARP4 were respectively determined by qRT-PCR analysis and western blot assays. **d** Luciferase reporter assay was used to test the luciferase activity of LARP4 promoter region. **e** qRT-PCR analysis and western blot assays were applied to analyze the mRNA and protein expression of LARP4 in different groups. **f** The proliferation ability of transfected cells was measured via colony formation. **g** Cell invasion capability in different groups was evaluated via transwell. **h** Western blot analysis of migration-related proteins was conducted. Results were revealed as the mean ± SD. **P < 0.01

And then we investigated the connection between circ_LARP4 and its host gene LARP4. As illustrated from Fig. 3a, subcellular fractionation assay showed that circ_LARP4 was mainly located in cytoplasm of SKOV3 and A2780 cells. Data from FISH assay further confirmed the fact (Fig. 3b). They both exhibited that circ_LARP4 could serve as a competing endogenous RNA (ceRNA) in OC cells. Additionally, as illustrated from Fig. 3c and Additional file 2: Figure S2C, upregulated circ_LARP4 conspicuously elevated the mRNA and protein expression of LARP4. Subsequently, luciferase reporter assay showed that the luciferase activity of LARP4 promoter region presented no significant difference after overexpressing circ_LARP4 (Fig. 3d). It further validated that LARP4 didn’t engage in regulating transcription. To investigate the biological effect of LARP4 on OC progression, LARP4 expression was elevated in SKOV3 and A2780 cells by transfection with OE-LARP4 at first (Fig. 3e, Additional file 2: Figure S2D). Then, data from colony formation assay revealed that LARP4 upregulation could suppress cell proliferation (Fig. 3f). Likewise, upregulating LARP4 resulted in attenuated capability of SKOV3 and A2780 cells to invade (Fig. 3g). Moreover, the expression of migration-related proteins (MMP2, MMP7 and MMP9) was notably decreased after LARP4 was overexpressed in SKOV3 and A2780 cells, indicating the suppressive effect of upregulated LARP4 on cell metastasis (Fig. 3h, Additional file 2: Figure S2E). All in all, circ_LARP4 posttranscriptionally regulates LARP4 expression and LARP4 upregulation exerts restraining effect on OC progression.

### Circ_LARP4 works as a sponge for miR-513b-5p to modulate LARP4

**Fig. 4:**
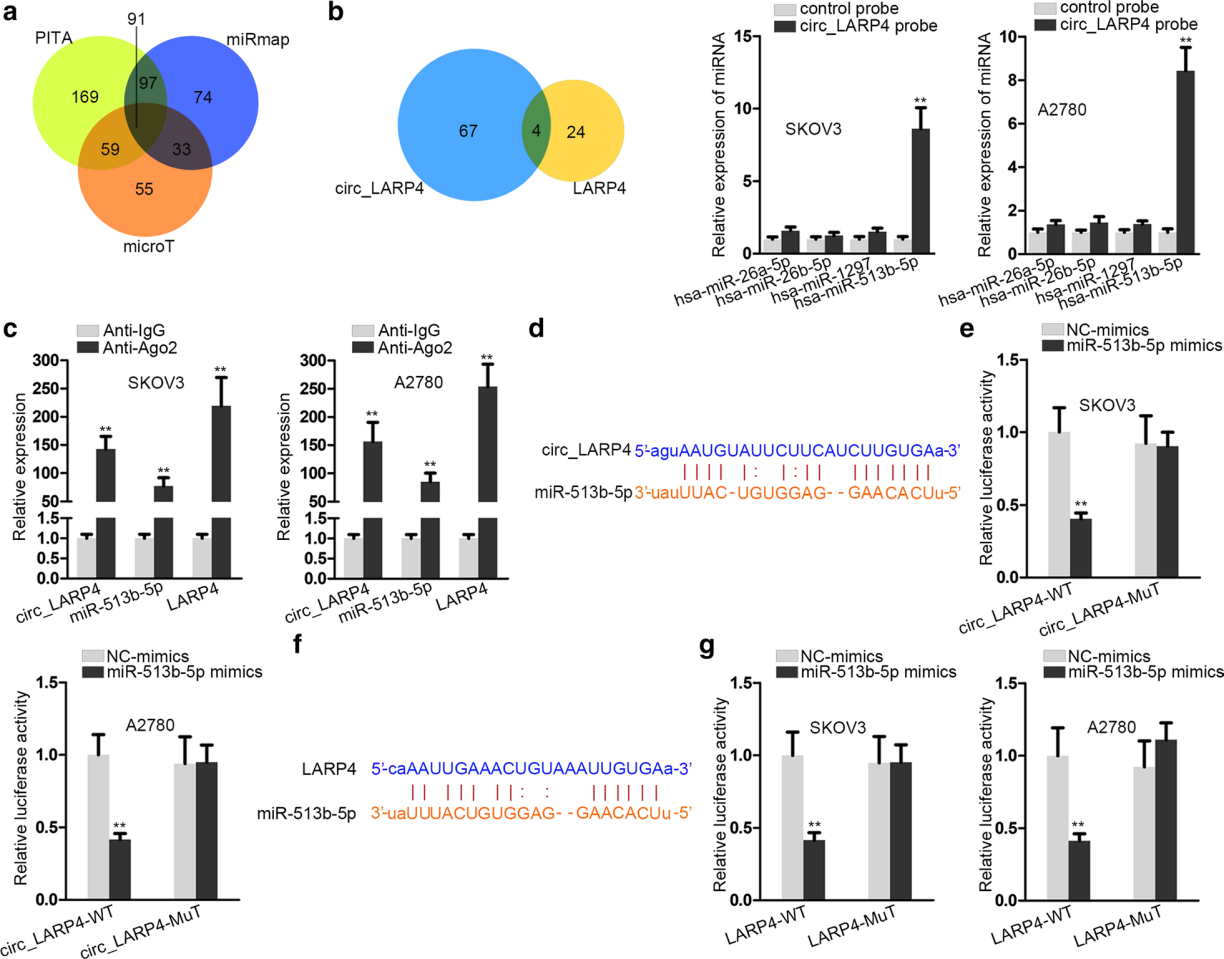
Circ_LARP4 works as a sponge for miR-513b-5p to modulate LARP4. **a** Three websites (PITA, microT and miRmap) were utilized to predict the potential miRNAs which could bind to LARP4. **b** Venn diagram was used to screen out the target miRNA which could bind to circ_LARP4 and LARP4 simultaneously. RNA pull down assay and qRT-PCR tested the expression of candidate RNAs. **c** RNA immunoprecipitation (RIP) assay was utilized to verify that circ_LARP4, miR-513b-5p and LARP4 co-existed in RNA-induced-silencing-complex (RISC). **d** The potential binding sites between circ_LARP4 and miR-513b-5p were depicted by starBase website. **e** The binding relationship between circ_LARP4 and miR-513b-5p was attested by luciferase reporter assay. **f** The binding sites between LARP4 and miR-513b-5p were acquired from starBase website. **g** Luciferase reporter assay was performed to detect the binding correlation between LARP4 and miR-513b-5p. All data were showed as the mean ± SD. **P < 0.01

Based on the aforementioned findings that circ_LARP4 was mainly located in cytoplasm and circ_LARP4 elicited no obvious effect on LARP4 promoter, we predicted that circ_LARP4 might work as a sponge for miRNAs to posttranscriptionally regulate LARP4 expression. We screened out 91 miRNAs which could bind to LARP4 from three prediction websites, including PITA, microT and miRmap in Fig. 4a. Then we further singled out 4 candidate miRNAs which could bind to circ_LARP4 and LARP4 simultaneously. Through detecting the enrichment of 4 miRNAs pulled down by circ_LARP4 in OC cells, miR-513b-5p was determined on account of its relative strong binding capacity with circ_LARP4 (Fig. 4b). As depicted in Fig. 4c, RIP assay was utilized to display that the circ_LARP4, miR-513b-5p and LARP4 were all enriched in the beads containing anti-Ago2, which denoted that circ_LARP4, miR-513b-5p and LARP4 co-existed in RNA-induced-silencing-complex (RISC). Figure 4d exhibited the binding sites between circ_LARP4 and miR-513b-5p. In the meantime, luciferase reporter assay revealed that the luciferase activity of wild type circ_LARP4 (circ_LARP4-WT) group was cut down observably by transfection with miR-513b-5p mimics, while that of mutant type circ_LARP4 (circ_LARP4-MUT) had no difference by contrast (Fig. 4e). It validated the binding relationship between circ_LARP4 and miR-513b-5p. Similarly, Fig. 4f presented the binding sites between LARP4 and miR-513b-5p. As indicated in Fig. 4g, the luciferase activity of wild type LARP4 (LARP4-WT) was depleted predominantly by transfected with miR-513b-5p mimics, and that of mutant type LARP4 (LARP4-MUT) group bore little alteration. The result approved the binding relationship between LARP4 and miR-513b-5p. Taken together, circ_LARP4 sponges miR-513b-5p to indirectly modulate the expression of LARP4 in OC cells.

### Circ_LARP4 inhibits the development of OC by regulating miR-513b-5p/LARP4 axis

**Fig. 5:**
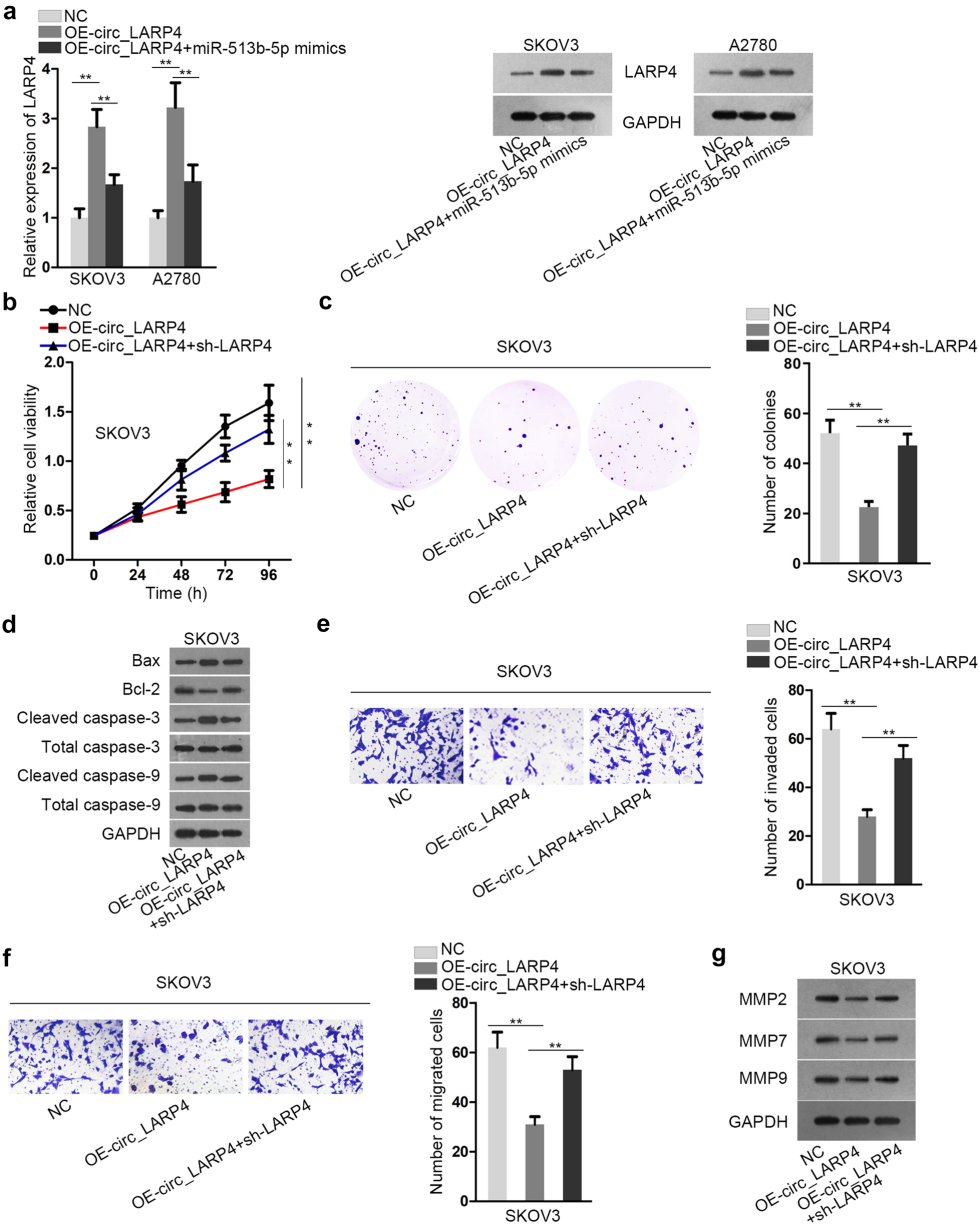
Circ_LARP4 inhibits the development of OC by regulating miR-513b-5p/LARP4 axis. **a** The qRT-PCR analysis and western blot assay were carried out to separately determine the expression and protein level of LARP4 in cells transfected with OE-circ_LARP4 and miR-513b-5p mimics. **b**, **c** CCK-8 assay and colony formation assay were performed to measure cell proliferation capacities. **d** Cell apoptosis-related protein levels were tested by western blot assay. **e**, **f** Transwell assay was carried out to estimate cell invasion and migration abilities. **g** The expression of proteins which were correlated to cell invasion and migration was measured by western blot analysis. All data were presented as the mean ± SD. **P < 0.01

In the last step, we applied rescue functional experiments to test the regulation effect of circ_LARP4/miR-513b-5p/LARP4 axis on OC cells. Just as uncovered in Fig. 5a and Additional file 2: Figure S2F, overexpressing miR-513b-5p rescued the facilitating effect of circ_LARP4 upregulation on LARP4 expression. It confirmed that circ_LARP4 modulated the expression of LARP4 by sponging miR-513b-5p. After we silenced LARP4 or overexpressed miR-513b-5p, the attenuated cell viability induced by upregulated circ_LARP4 was reversed (Fig. 5b, c, Additional file 1: Figure S1A, B). Inversely, sh-LARP4 or miR-513b-5p mimics could restore the promoting effect of circ_LARP4 upregulation on cell apoptosis. It could be seen from the alteration of apoptosis-related proteins expression (Fig. 5d, Additional file 1: Figure S1C, Additional file 2: Figure S2G, I). Data from transwell assay revealed that reducing LARP4 expression or overexpressing miR-513b-5p countervailed the restraining influence of OE-circ_LARP4 on cell invasion and migration (Fig. 5e, f, Additional file 1: Figure S1D, E). Further, the suppressive effect of circ_LARP4 overexpression on the expression of migration-related proteins could be rescued by silencing LARP4 or overexpressing miR-513b-5p (Fig. 5g, Additional file 1: Figure S1F, Additional file 2: Figure S2H, J). To conclude, circ_LARP4 impairs OC progression via miR-513b-5p/LARP4 axis.

## Discussion

LARP4 gene existed in some protists and all animals tested, while not in plants and yeasts [18]. LARP4 of mammal, which was also called LARP4A, had close interaction with poly (A) RNA. It indicated that LARP4 could bind to poly (A) tail. As a matter of fact, overexpressing LARP4 induces the increase of mRNA stability, whereas cutting down LARP4 leads to translation declined by 15 percent to 20 percent. It presented the promotion of LARP4 on mRNA stability [19]. Therefore, LARP4 could regulate cellular morphology through the bind with mRNA and translation. Besides, under the condition, LARP4 may specially correlated to the interaction of RACK1, because there has reporter said that RACK1 exerted important effect on cell adhesion and migration [20]. In this research, LARP4 worked as the host gene of circ_LARP4. The expression and protein level of LARP4 were apparently increased by overexpressing circ_LARP4. LARP4 acted as the target gene of miR-513b-5p, and had binding sites with miR-513b-5p. Furthermore, in the last rescue experiments, depleting LARP4 or overexpressing miR-513b-5p was able to recover cell viability, colony formation efficiency, invasion and migration capabilities which were impaired by increasing circ_LARP4. These results all proofed that LARP4 served as the host gene of circ_LARP4 and was modulated indirectly by circ_LARP4 in OC cells.

Mounting researches have depicted that circRNAs not only were wrong splicing secondary products, but also could regulate gene expression and fully acted as the sponge for miRNAs in pathogenesis of cancers [14, 21–23]. CircRNAs as tumor suppressors or oncogenes exerted crucial impact on cancer progression. For example, circCCDC66a and circ_0067934 promote tumor growth and metastasis [24, 25], while circZKSCAN1 and circZNF292 hampered cancer progression through multiple signal routings [26, 27]. Here, we assessed a circRNA derived from gene sites of LARP4, which called circ_LARP4. We found that circ_LARP4 had lower expression in OC cell lines compared with the normal ovarian epithelial cell. In addition, circ_LARP4 had stronger stability and less susceptibility than LARP4 in OC cell lines. Besides, overexpressing circ_LARP4 hampered cell active abilities, including cell proliferation, migration and invasion, whereas promoted cell apoptosis. These findings determined that circ_LARP4 played a tumor-suppressing role in OC.

## Conclusion

In this study, we applied starBase v2.0 to forecast the potential miRNAs which could bind with circ_LARP4 and LARP4. Within the candidate miRNAs, we singled out miR-513b-5p owing to its strong binding capacity with LARP4 in OC cells. On the one hand, circ_LARP4 could sponge miR-513b-5p through binding with it. On the other hand, LARP4 acting as the target gene of miR-513b-5p also shared binding sites with miR-513b-5p. Furthermore, up-regulating miR-513b-5p could rescue the effect of upregulated circ_LARP4 on LARP4 expression, confirming that LARP4 expression was regulated by circ_LARP4 via sponging miR-513b-5p. To draw a conclusion, circ_LARP4 suppresses the progression of OC via miR-513b-5p/LARP4 axis (Graphical Abstract). The finding of circ_LARP4/miR-513b-5p/LARP4 pathway provides evidence of the suppressive function of circ_LARP4 on OC progression together with its ceRNA mechanism in OC, providing novel therapeutic targets for OC treatments. Further clinical investigations are the future direction for our study.

## Supplementary information

**Additional file 1: Figure S1.** A-B. Cell proliferation capacity was analyzed via CCK-8 assay and colony formation assay. C. Cell apoptosis-related protein levels were tested by western blot assay. D-E. Transwell assay was conducted to estimate cell invasion and migration abilities. F. The expression of metastasis-related proteins was measured via western blot analysis. All data were presented as the mean ± SD. **P < 0.01.

**Additional file 2: Figure S2.** A-G. Quantification of western blot analyses. GAPDH was an internal control. All data were presented as the mean ± SD. **P < 0.01.

## Data Availability

Research data and material are not shared.

## Abbreviations

OC: ovarian cancer
HuO: human osteosarcoma
circRNA: circular RNA
circ_LARP4: circular RNA_LARP4
miRNA: microRNA
RBP: RNA binding protein
mRNA: messenger RNA
FISH: fluorescence in situ hybridization
ceRNA: competing endogenous RNA
RISC: RNA-induced silencing complex
circ_LARP4-WT: wild type circ_LARP4
circ_LARP4-MUT: mutant type circ_LARP4
LARP4-WT: wild type LARP4
LARP4-MUT: mutant type LARP4
OE-circ_LARP4: circ_LARP4 overexpression
CCK-8: cell counting kit-8
RIP: RNA immunoprecipitation
qRT-PCR: quantitative real-time polymerase chain reaction
