# Supplementary figures and images for: Up-regulation of circ_LARP4 suppresses cell proliferation and migration in ovarian cancer by regulating miR-513b-5p/LARP4 axis
Source: Cancer Cell Int. 2020 Jan 6;20:5. doi: 10.1186/s12935-019-1071-z (PMC6945592; doi:10.1186/s12935-019-1071-z)

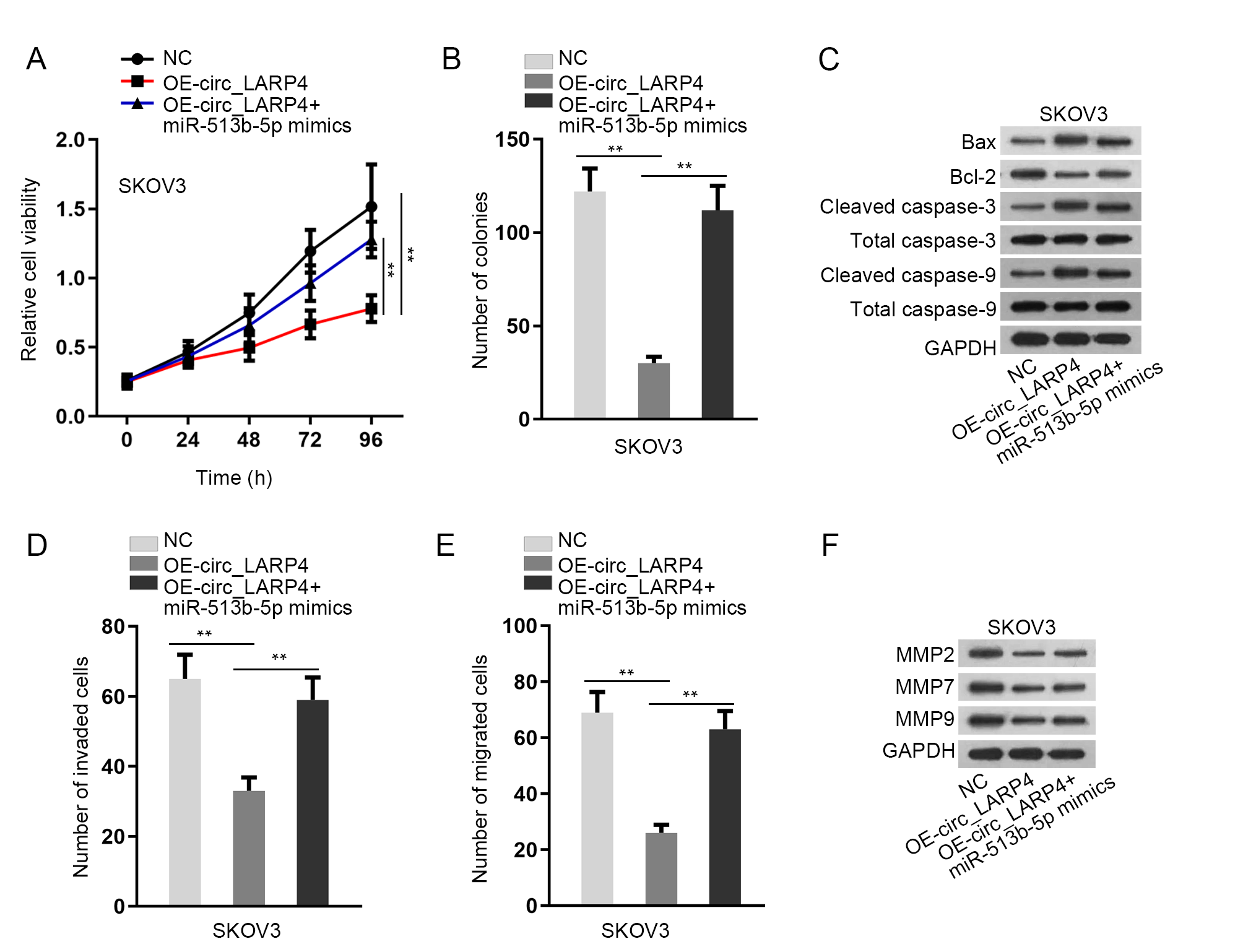

Supplement: Supplementary file 1 — Additional file 1: Figure S1. A-B. Cell proliferation capacity was analyzed via CCK-8 assay and colony formation assay. C. Cell apoptosis-related protein levels were tested by western blot assay. D-E. Transwell assay was conducted to estimate cell invasion and migration abilities. F. The expression of metastasis-related proteins was measured via western blot analysis. All data were presented as the mean ± SD. **P < 0.01. [file 12935_2019_1071_MOESM1_ESM.tif]

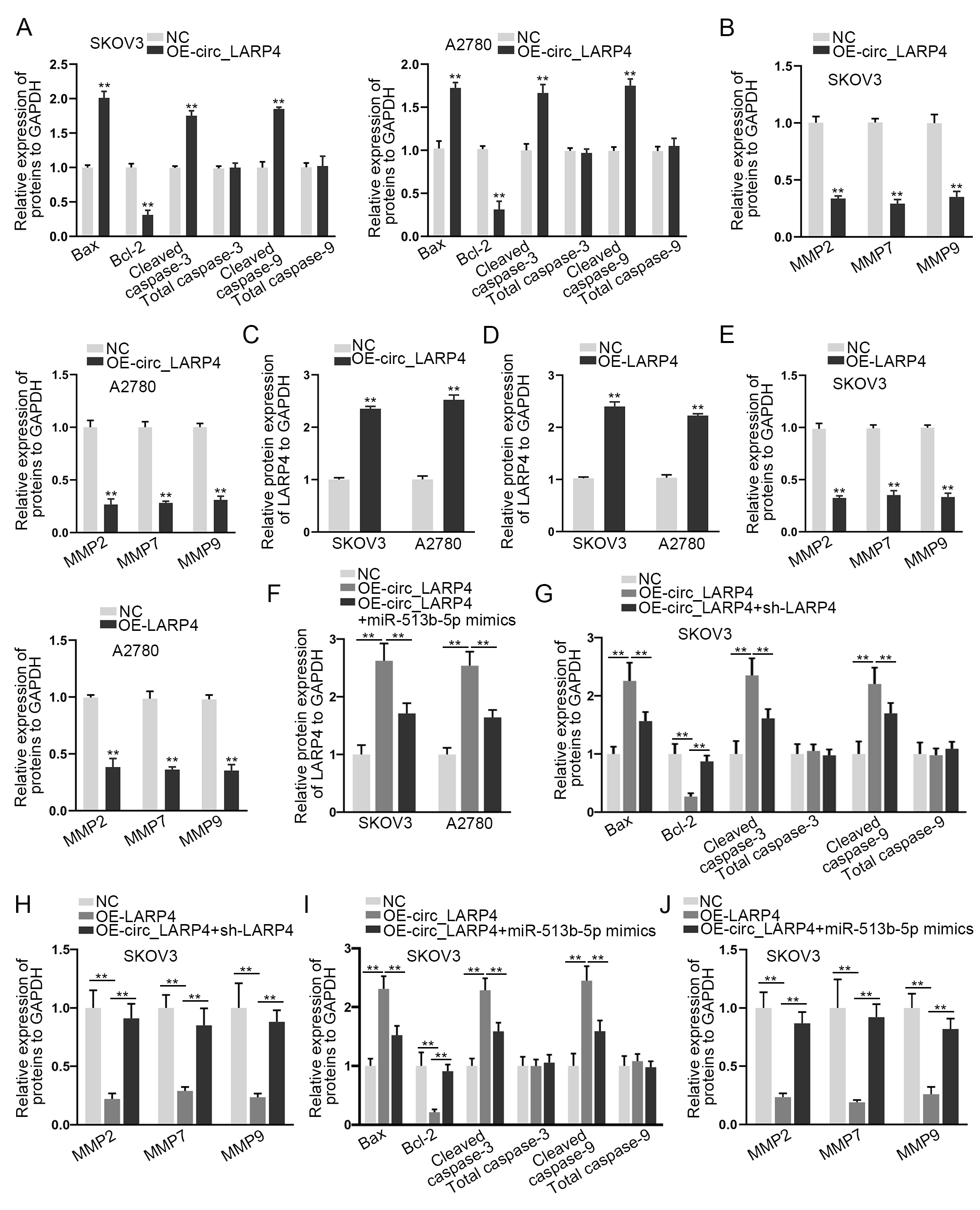

Supplement: Supplementary file 2 — Additional file 2: Figure S2. A-G. Quantification of western blot analyses. GAPDH was an internal control. All data were presented as the mean ± SD. **P < 0.01. [file 12935_2019_1071_MOESM2_ESM.tif]
